# Correlation Between External and Internal Skin Aging Markers by Skin Depth

**DOI:** 10.1111/jocd.70354

**Published:** 2025-07-31

**Authors:** Hye‐Yeon Yang, Sung‐Ha Park, Ji‐Eun Woo, Byoung‐Jun Park

**Affiliations:** ^1^ Skin&Natural Products Lab Kolmar Korea Co. Ltd. Seoul Republic of Korea

**Keywords:** dermis, epidermis, external skin markers, internal skin markers, skin aging, skin depth

## Abstract

**Background:**

The skin is a complex, multilayered organ with diverse structures. Skin aging is a gradual process that begins internally, initially invisible to the eye, and eventually manifests as visible signs such as wrinkles and sagging.

**Aims:**

To investigate the effects of characteristic changes in different skin layers on visible signs of aging, such as wrinkles and sagging.

**Methods:**

A total of 65 Korean women aged 20–65 years participated in this study. Skin characteristics were categorized into visible external and invisible internal markers. External markers included wrinkles and sagging, whereas internal markers included hydration, elasticity, density, thickness, and dermal‐subcutaneous interface length. Internal markers were measured in greater detail at various skin depths. Spearman's correlation was used to analyze relationships between all skin parameters, including age.

**Results:**

Visible signs of aging such as wrinkles and sagging increased with age. The elasticity indicators in each skin layer tended to decrease. No correlation was observed between moisture levels and age. The results of the correlation analysis between external and internal markers varied according to skin layer. Negative correlations were observed between skin sagging and epidermal moisture content in the epidermis and between wrinkles and elasticity indicators (Ue and Ur) in the deeper dermis layer.

**Conclusions:**

External markers are most strongly correlated with chronological age. However, visible aging is significantly influenced by changes in internal markers across different skin layers. Sagging is affected by moisture content within the epidermal layer, and wrinkles are affected by elasticity within the dermal layer.

## 1 Introduction

The skin is a large, complex organ with multiple layers that differ in structure and function. It forms the outermost layer of the body and serves as a barrier between the external environment and the body's interior [1]. Over time, the skin undergoes functional and structural changes [2], and the causes of this aging can be broadly categorized into external and internal factors [3, 4, 5].

Exogenous aging refers to aging caused by external environmental factors, such as ultraviolet radiation, infrared radiation, and air pollution. The most prominent form of exogenous aging is photoaging, which is caused by ultraviolet radiation. Symptoms of photoaging include decreased skin elasticity, increased wrinkles, and pigmentation [6]. Recent studies have shown that wrinkles, pigmentation, and sagging are more pronounced in older individuals due to their prolonged exposure to sunlight over time [7].

Endogenous aging refers to the natural changes in tissue that occur over time due to genetic factors and the aging process [4, 8]. The key characteristics of skin aging include increased skin surface roughness, wrinkle formation, and subepidermal atrophy [9]. Specifically, as we age, the amount of hyaluronic acid in the epidermis decreases [10], which can lead to reduced skin hydration, elasticity, and smoothness [11]. Simultaneously, the dermis experiences a decline in collagen and elastic fibers, diminishing the skin's elasticity and resilience, which further contributes to wrinkle formation [12].

Skin aging progresses gradually from within, starting with invisible internal changes that eventually manifest as visible features on the skin's surface, such as wrinkles and sagging. These characteristics are considered classic markers of aging and typically become more pronounced as individuals grow older [13].

Aging exhibits different patterns depending on gender. Collagen in the dermis decreases more rapidly in women with age, resulting in thinner skin compared to men [14]. Dermal elastin also shows a more pronounced decrease in women compared to men [15], and skin elasticity decreases by 1.5% per year in the first 5 years after menopause [16, 17].

Until recently, anti‐aging treatments have primarily focused on managing visible signs of aging, such as wrinkles and sagging around the eyes. However, in recent years, there has been a growing interest in “early anti‐aging,” which aims to prevent and manage aging proactively, in addition to improving already aged skin. Despite this shift, there is still no definitive marker to identify internal skin aging before visible signs appear on the skin's surface.

Therefore, this study aimed to investigate the effects of characteristic changes in different skin layers on visible signs of aging, such as wrinkles and sagging. The findings can provide valuable insights into developing strategies for managing aging before it becomes apparent.

## 2 Materials and Methods

### 2.1 Participants

Sixty‐five Korean women aged 20–65 years participated in this study. The study was approved by the Dermapro Institutional Ethics Committee (approval number: 12207777‐A‐N‐01‐DICN22280) and conducted in accordance with the basic principles of the Declaration of Helsinki. Informed consent was obtained from all participants after providing a complete explanation of the protocol.

### 2.2 Skin Biophysical Measurements

This study was conducted after a 20min stabilization period following facial cleansing under controlled temperature (22°C ± 2°C) and humidity (50% ± 5%) conditions. The test site was selected as the crow's feet area, and all skin parameters were measured using noninvasive methods.

#### 2.2.1 External Skin Markers

Wrinkles and sagging are the most common visible signs of aging that appear on the skin's surface. These characteristics are categorized as external skin markers. Skin surface wrinkles were measured using PRIMOS‐CR (GFMesstechnik GmbH, Berlin, Germany), and the representative parameters Ra, Rt, and Rz were analyzed. The skin sagging angle was measured using F‐ray (Beyoung, Seoul, Korea) and analyzed using Image‐Pro 10 (Media Cybernetics, Rockville, MD, USA) on facial contour images. The measuring devices for each skin characteristic are listed in Table 1.

**TABLE 1:** Instruments and parameters for measuring external skin markers.

External skin markers	Parameter	Unit	Instruments
Wrinkle	Ra (arithmetic mean roughness)	μm	PRIMOSCR (GFMesstechnik GmbH, Germany)
	Rt (maximum peak to valley height)	μm	
	Rz (mean roughness depth)	μm	
Sagging	Angle	°	F‐ray (Beyoung, Korea)

#### 2.2.2 Internal Skin Markers

Skin characteristics that are not visible on the surface and cannot be observed with the naked eye are classified as internal skin markers. These include skin hydration, elasticity, epidermal and dermal thickness, skin density, and dermal‐subcutaneous tissue interface length. Among these, skin hydration and elasticity were measured at various depths to understand their characteristics at different levels within the skin.

Skin hydration was measured at four depths using each probe of the Moisturemeter D (Delfin, Kuopio, Finland). The 0.5 and 1.5 mm depths were considered epidermal, whereas the 2.5 and 5 mm depths were considered dermal. Skin elasticity was evaluated using the Dermal Torque Meter DTM310 (Dia‐Stron, Broomall, PA, USA) with 1‐ and 3‐mm probes to assess skin elasticity at superficial and deep levels, respectively. The 1 mm depth measurement represented the elasticity of the epidermis, whereas the 3 mm depth measurement reflected the elasticity of the full skin thickness, including both the epidermis and dermis. Skin thickness, skin density, and dermal‐subcutaneous tissue interface length were measured using a DUB Skin Scanner (50 MHz) (tpm GmbH, Lueneburg, Germany). Skin thickness was evaluated separately for the epidermis and dermis. The measuring devices and depths for each skin characteristic are shown in Table 2.

**TABLE 2:** Instruments and parameters for measuring internal skin markers.

Internal skin marker	Parameter	Unit	Depth				Instruments
			Epidermis		Dermis		
Hydration	Skin moisture content	AU	0.5 mm	1.5 mm	2.5 mm	5 mm	Moisturemeter D (Delfin, Finland)
Elasticity	Ue (immediate extensibility)	AU	1 mm		3 mm		Dermal Torque Meter DTM310 (Dia‐Stron, USA)
	Uv (additional extensibility)	AU					
	Ur (immediate recovery)	AU					
	Uf (total extensibility)	AU					
	R7 (skin recovery elasticity)	AU					
	R5 (instant elastic recovery)	AU					
Density	Density	Density	~3 mm				DUB Skin Scanner (50 MHz) (tpm GmbH, Germany)
Thickness	Thickness	μm	~1 mm		~3 mm		
Dermis‐subcutaneous tissue length	Length	mm	—				

### 2.3 Statistical Analysis

Statistical analysis was performed using SPSS software 24.0 for Windows (IBM SPSS, Armonk, NY, USA). Spearman's correlation coefficient (*ρ*, also signified by rs) measured the strength and direction of association between two ranked variables. Spearman's coefficients were calculated, and a correlation was considered statistically significant if the absolute value of the correlation coefficient was between 0.3 and 0.8 and the *p*‐value was < 0.05. The sign of a coefficient represents the direction of the relationship. If two variables tend to increase or decrease together, the coefficient is positive, and the line representing the correlation slopes upward. If one variable increases while the other tends to decrease, the coefficient is negative, and the line representing the correlation slopes downward.

## 3 Results

### 3.1 Correlation Between Age and Skin Markers

**FIGURE 1:**
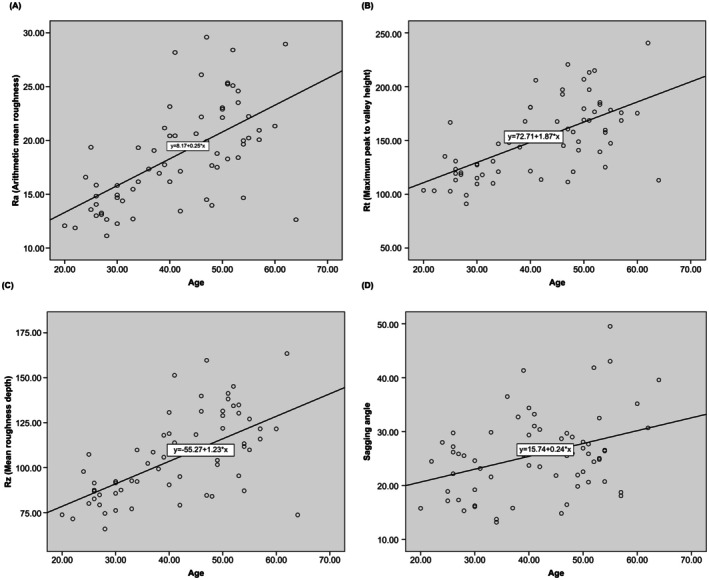
Correlation between age and skin wrinkles (Ra, Rt, and Rz) and sagging. (A–C) Skin wrinkles (Ra, Rt, and Rz); (D) Skin sagging angle.

**FIGURE 2:**
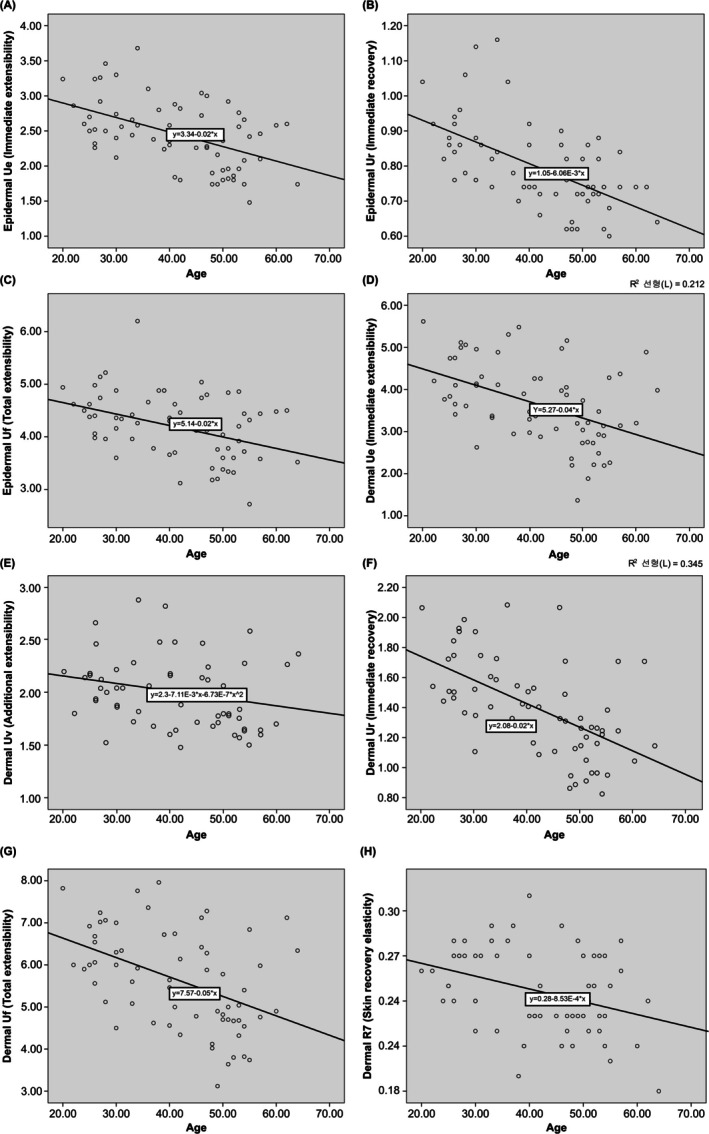
Correlation between skin markers by age and depth. (A–C) Epidermal elasticity (Ue, Ur, and Uf); (D–H) dermal elasticity (Ue, Uv, Ur, Uf, R7).

The external markers of visible aging, such as skin wrinkles (Ra, Rt, and Rz) and skin sagging, showed a significant positive correlation with age (Table 3, Figure 1) (Ra, *R* = 0.616, *p* < 0.001; Rt, *R* = 0.605, *p* < 0.001; Rz, *R* = 0.599, *p* < 0.001; sagging angle, *R* = 0.318, *p* = 0.01). In other words, skin wrinkles and sagging increase with age. Meanwhile, among the internal skin markers, certain skin elasticity measures tended to decrease with age (Table 4, Figure 2). Elasticity markers, including immediate elasticity (Ue), immediate recovery (Ur), and total extensibility (Uf), significantly decreased across both the epidermis and dermis layers. Particularly, Ur exhibited the strongest correlation with age. Conversely, R7, which represents skin recovery elasticity, showed a negative correlation with age only in the dermis layer. Except for skin elasticity, no other internal skin markers were correlated with age, regardless of the skin layer.

**TABLE 3:** Spearman's correlation coefficient showing an evaluation of the relationship between age and external and skin markers.

	Parameter	Age	*p*
Wrinkle	Ra (arithmetic mean roughness)	0.616**	0.00
	Rt (maximum peak to valley height)	0.605**	0.00
	Rz (mean roughness depth)	0.599**	0.00
Sagging	Angle	0.318**	0.01

** *p* < 0.05 by Spearman's correlation.

**TABLE 4:** Spearman's correlation coefficient showing the relationship between age and internal skin markers.

	Depth (mm)	Parameter	Age	*p*
Hydration	0.5	Skin moisture content	−0.166	0.186
	1.5		−0.132	0.296
	2.5		−0.213	0.089
	5		−0.153	0.223
Elasticity	1	Ue (immediate extensibility)	−0.479**	0.00
		Uv (additional extensibility)	0.03	0.812
		Ur (immediate recovery)	−0.599**	0.00
		Uf (total extensibility)	−0.415**	0.001
		R7 (skin recovery elasticity)	−0.173	0.167
		R5 (instant elastic recovery)	0.156	0.216
	3	Ue (immediate extensibility)	−0.471**	0.00
		Uv (additional extensibility)	−0.308*	0.013
		Ur (immediate recovery)	−0.626**	0.00
		Uf (total extensibility)	−0.460**	0.00
		R7 (skin recovery elasticity)	−0.324**	0.008
		R5 (instant elastic recovery)	−0.056	0.66
Dermal density			0.15	0.233
Epidermal thickness			0.123	0.327
Dermal thickness			−0.023	0.853
Dermis‐subcutaneous tissue length			−0.019	0.881

* *p* < 0.1 by Spearman's correlation.

** *p* < 0.05 by Spearman's correlation.

### 3.2 Correlation Between Internal Markers by Skin Layer and External Markers Representing Visible Aging

**FIGURE 3:**
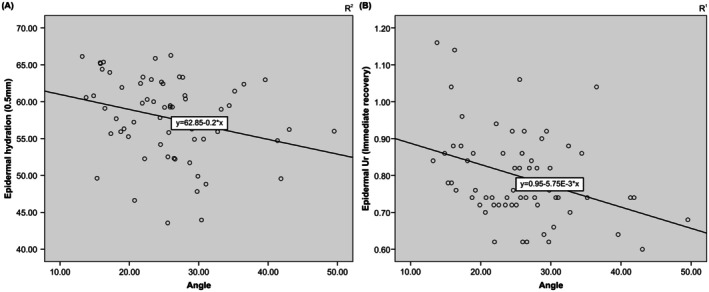
Correlation between skin sagging and skin epidermal markers. (A) Skin epidermal hydration; (B) epidermal Ur (immediate recovery).

**FIGURE 4:**
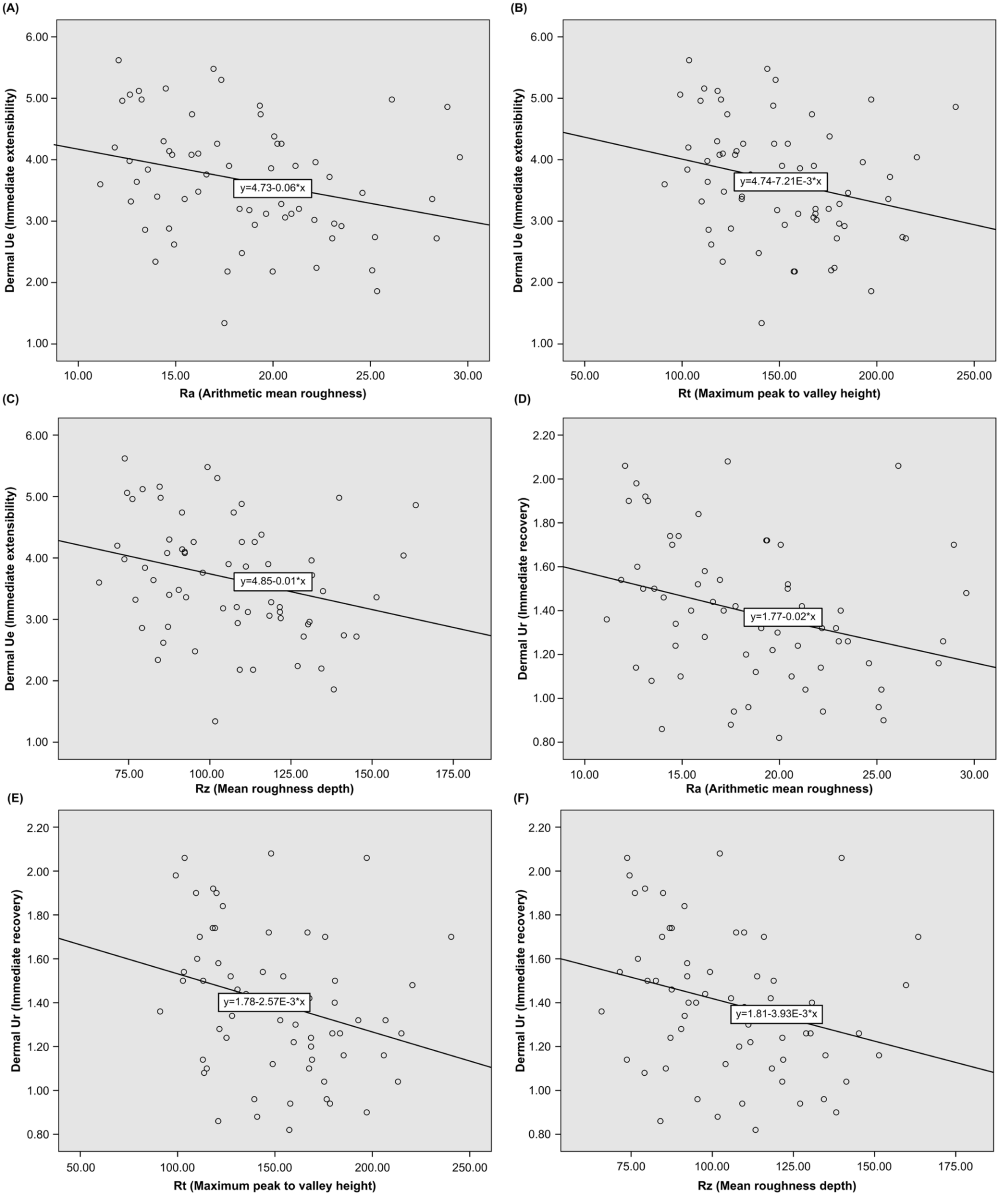
Correlation between skin wrinkles (Ra, Rt, and Rz) and skin dermis markers. (A–C) Skin dermis Ue (immediate extensibility); (D–F) Skin dermis Ur (immediate recovery).

A correlation analysis was performed between external skin markers and internal markers by skin layer, excluding age as a variable, to investigate the effects of internal markers from different skin layers on external markers representing visible signs of aging. Skin sagging was negatively correlated with epidermal moisture content and immediate recovery (Ur) (Table 5, Figure 3). No correlations were observed between skin sagging and any other internal markers in the epidermal layer or any internal markers in the dermal layer. Negative correlations were observed between external markers representing skin wrinkles (Ra, Rt, and Rz) and Ue (immediate extensibility) and Ur (immediate recovery) values at a depth corresponding to the dermal layer (3 mm) (Table 5, Figure 4). Additionally, no correlations were observed between skin wrinkles and other internal markers in the dermal layer, including dermal moisture content, or any internal markers in the epidermal layer.

**TABLE 5:** Spearman's correlation coefficient showing the relationship between external skin markers and internal skin markers.

	Depth	Parameter	Sagging		Ra		Rt		Rz	
			Spearman's correlation coefficient	*p*	Spearman's correlation coefficient	*p*	Spearman's correlation coefficient	*p*	Spearman's correlation coefficient	*p*
Hydration	0.5	Skin moisture content	−0.337**	0.006	−0.093	0.462	−0.06	0.637	−0.091	0.472
	1.5		−0.256*	0.039	−0.141	0.263	−0.106	0.403	−0.139	0.268
	2.5		−0.242	0.053	−0.146	0.244	−0.109	0.388	−0.147	0.242
	5		−0.270*	0.03	−0.162	0.199	−0.133	0.291	−0.166	0.187
Elasticity	1	Ue (immediate extensibility)	−0.225	0.071	−0.308*	0.013	−0.283*	0.023	−0.288*	0.02
		Uv (additional extensibility)	0.003	0.982	0.176	0.16	0.203	0.104	0.179	0.153
		Ur (immediate recovery)	−0.318**	0.01	−0.288*	0.02	−0.274*	0.027	−0.260*	0.037
		Uf (total extensibility)	−0.149	0.237	−0.207	0.098	−0.174	0.166	−0.189	0.131
		R7 (skin elasticity)	−0.138	0.272	−0.101	0.425	−0.117	0.354	−0.088	0.485
		R5 (elastic recovery)	0.04	0.754	0.146	0.245	0.122	0.332	0.145	0.25
	3	Ue (immediate extensibility)	−0.132	0.293	−0.332**	0.007	−0.324**	0.009	−0.316*	0.01
		Uv (additional extensibility)	0.059	0.643	−0.118	0.348	−0.112	0.376	−0.113	0.372
		Ur (immediate recovery)	−0.228	0.068	−0.355**	0.004	−0.342**	0.005	−0.336**	0.006
		Uf (total extensibility)	−0.072	0.566	−0.289*	0.02	−0.280*	0.024	−0.274*	0.027
		R7 (skin elasticity)	−0.281*	0.023	−0.176	0.161	−0.16	0.204	−0.166	0.187
		R5 (elastic recovery)	−0.125	0.319	0.017	0.896	0.022	0.859	0.022	0.863
Dermal density					−0.088	0.484	−0.001	0.996	0.033	0.793
Epidermal thickness					−0.046	0.719	0.077	0.54	0.123	0.33
Dermal thickness					0.072	0.569	0.141	0.264	0.145	0.248
Dermis‐subcutaneous tissue length					−0.034	0.791	0.101	0.421	0.07	0.579

* *p* < 0.1 by Spearman's correlation.

** *p* < 0.05 by Spearman's correlation.

## 4 Discussion

The significance of this study lies in that, unlike previous clinical studies that considered the skin as a single layer and focused solely on surface characteristics, it categorizes the skin into layers based on depth and analyzes factors within each layer that contribute to visible signs of aging.

Representative signs of skin aging include increased roughness and sagging [18, 19, 20], decreased brightness (*L* value) [21, 22], and reduced elasticity and elastic recovery [23, 24]. Although various factors influence skin aging, age is the most strongly correlated factor. Recently, several attempts have been made to investigate the correlations between skin properties to identify factors interacting with skin aging beyond age [25]. However, previous studies have only examined correlations between surface‐level skin characteristics.

This study showed a strong correlation between representative signs of aging, such as wrinkles (Ra, Rt, and Rz) and sagging, and age. Among the internal markers of skin layers, certain indicators of skin elasticity (Ue, Ur, and Uf) showed a tendency to decrease with age across all depths of the epidermis and dermis. In addition, R7 (Skin Recovery Elasticity) was negatively correlated with age only at dermal depth. These findings indicate skin hardening caused by the stiffening of keratin fibers in the stratum corneum and collagen fibers in the dermis, along with a reduction in resilience as age increases [13].

The correlation analysis between visible signs of aging and internal markers by skin layer indicated that skin sagging was negatively correlated with epidermal moisture content and immediate recovery (Ur). However, since the epidermal moisture content was not significantly correlated with age, a decrease in the epidermal moisture content might have a greater impact on the increase in sagging. Skin wrinkles (Ra, Rt, and Rz) were negatively correlated with immediate deformation and recovery elasticity at a depth corresponding to the dermis (3 mm). This finding indicates that the elasticity of the dermis may influence the formation of visible wrinkles.

Previous studies have primarily explained visible signs of aging, such as wrinkles and sagging, in relation to dermal aging. Building upon and extending this perspective, this study highlights the potential role of epidermal aging in contributing to sagging. By categorizing internal skin markers based on measurement depth and analyzing their correlations with external skin markers, this study provides valuable insights into the early detection of skin aging beyond conventional surface‐focused assessments. Unlike previous studies that primarily regarded the skin as a single‐layered structure or focused solely on surface‐level characteristics, this study underscores the importance of depth‐specific variations in internal markers and their influence on external aging markers. By employing a layered analytical approach, this study provides a novel perspective on skin aging, advancing beyond traditional surface‐focused methodologies.

This study has some limitations. First, this study employed a cross‐sectional design, which did not allow for longitudinal tracking of individual skin condition changes over time. Therefore, further longitudinal studies are needed to better capture the dynamics of aging at the individual level. Second, this study focused exclusively on Korean female participants, which limits the generalizability of the results to other genders or racial groups. Therefore, further studies including more diverse populations are needed to enhance the external validity of the findings. Although this study focused on the quantitative correlation between internal markers and external features, a more comprehensive understanding of skin aging could be achieved by analyzing biochemical components such as collagen, elastin, and hyaluronic acid. Particularly, the dermal–epidermal junction (DEJ), which plays a critical role in mechanical stability and signal transmission between the epidermis and dermis, should be studied in relation to aging. Investigating age‐related changes in DEJ‐associated proteins, such as laminin, integrin, and fibronectin, can provide insights into how the degradation of this interface contributes to external signs of aging.

## Author Contributions

Hye‐Yeon Yang: Conceptualization, Methodology, Software, Validation, Formal Analysis, Investigation, Resources, Writing – Original Draft Preparation, Writing – Review and Editing; Ji‐Eun Woo: Conceptualization, Methodology, Validation, Formal Analysis, Investigation; Byoung‐Jun Park: Data Curation, Visualization, Supervision, Project Administration; Sung‐Ha Park: Data Curation, Visualization, Supervision, Project Administration. All authors have read and approved the final version of the manuscript for publication.

## Ethics Statement

The study was approved by the Dermapro Institutional Ethics Committee (approval number: 12207777‐A‐N‐01‐DICN22280) and conducted in accordance with the basic principles of the Declaration of Helsinki. Informed consent was obtained from all participants after providing a complete explanation of the protocol.

## Conflicts of Interest

The authors declare no conflicts of interest.

## Data Availability

All data generated or analyzed during this study are included in this published article.
